# Health professional’s knowledge and use of the partograph in public health institutions in eastern Ethiopia: a cross-sectional study

**DOI:** 10.1186/s12884-017-1477-3

**Published:** 2017-09-06

**Authors:** Haymanot Mezmur, Agumasie Semahegn, Balewgizie Sileshi Tegegne

**Affiliations:** 1 School of Nursing and Midwifery, College of Health and Medical Sciences, Haramaya University, Harar, Ethiopia; 2 Department of Public Health, College of Health and Medical Sciences, Haramaya University, Po. Box, 235, Harar, Ethiopia; 3 Department of Epidemiology, University of Groningen, University Medical Center Groningen, Groningen, the Netherlands

**Keywords:** Knowledge, Partograph, Health professionals, Utilization, Ethiopia

## Abstract

**Background:**

The partograph is a vital tool for health professionals who need to be able to identify pathological labor. It is used to recognize complications in childbirth on time and to take appropriate actions. We aimed to assess the knowledge and utilization of the partograph and associated factors among health professionals at public health institutions in eastern Ethiopia.

**Methods:**

An institution based cross-sectional quantitative study was carried out among health professionals who were working in public health institutions. Multistage sampling with proportional to size allocation was used to recruit a total of 441 study participants. Self-administered questionnaire was used to collect data in this study. Eight midwives were recruited and trained to facilitate the data collection activities. Data were entered into Epi data software and exported into SPSS (22.0) for analysis. Descriptive statistics, bivariate and multiple logistic regression were computed to determine proportions and significant association with knowledge and use of the partograph among health professionals.

**Results:**

More than half of health professionals, 232(53.7%) had a good level of knowledge about the partograph. However, only 196(45.4%) of health professionals had fair knowledge of partograph. Nevertheless, the proportion of the partograph utilization to follow labor progress by health professionals was 92.6%. Working in the health center [AOR = 0.31, 95% CI: 0.20, 0.48], being a midwife [AOR = 2.80, 95% CI: 1.60, 5.60] and in-service training [AOR = 2.0, 95% CI: 1.22, 3.42] were significantly associated with good level of knowledge. Health professionals who had in-service training about the partograph [AOR = 3.10, 95% CI: 1.35, 4.98] and who had positive attitude about the partograph [AOR = 2.90, 95% CI: 1.30, 6.30] were significantly associated with utilization of the partograph.

**Conclusion:**

Only less than half of health professionals had fair knowledge about the partograph. Having in-service obstetric care training, type of health institutions and profession were significantly associated with knowledge of the partograph. Health professionals who had positive attitude towards use of the partograph were significantly associated with the partograph utilization. We suggest regular in-service training of health professionals can enhance their knowledge and utilization of the partograph.

## Background

In the year 2015 alone, more than 300,000 maternal deaths occurred worldwide from complications during pregnancy or childbirth. Almost all of them (99%) were in developing countries of which Sub-Saharan Africa alone has accounted about 66% of deaths [1]. Woman’s life time risk of dying from preventable or treatable complications of pregnancy and childbirth in Sub-Saharan Africa was 1 in 31 as compared with only 1 in 4300 in the developed regions [2]. The Ethiopian demographic and health survey 2000 showed that maternal mortality ratio was 871 deaths per 100,000 live births in Ethiopia [3]. A slight reduction observed in 2005 and 2011 with 673 and 676 deaths per 100,000 live births, respectively [4, 5]. Despite the reduction of maternal mortality ratio was expected, it remains unchanged between 2005 and 2011. Moreover, there is no evidence to suggest that the maternal mortality ratio in Ethiopia decreased between 2000 and 2011 [5]. The majority of maternal deaths and complications are attributed to obstructed and prolonged labor. These can be prevented by using the partograph which is cost-effective and affordable health intervention [6, 7]. A partograph is a tool used to monitor labor and prevent prolonged and obstructed labor focusing on observations related to maternal, fetal condition and labor progress. The World Health Organization (WHO) partographs are the best known and most widely used in developing countries with the objective of reducing feto-maternal morbidity and death [7, 8]. According to WHO multicenter trial, the partograph introduced into clinical practice along with a management protocol labor outcomes were greatly improved. It facilitates need of augmentation of labor with utrotonics, caesarean section, and minimize incidence of infection. So WHO recommend to use the partograph for monitoring of all labors which helps for the early identification of abnormal progress of labor and prompt interventions [9].

The partograph has clear demarcations which clearly indicate the need to address existing or imminent complications like poor progress of labor, prolonged labor, fetal distress, obstructed labor and ruptured uterus. Early detection of prolonged or obstructed labor greatly contributes to the prevention of complications such as postpartum haemorrhages, ruptured uterus, puerperal sepsis and obstetric fistula [9]. The partograph provides a pictorial overview of the labor to health professionals that allow early identification and diagnosis of the pathological labor [9, 10]. Prolonged labor is a leading cause of death among mothers and newborns in the developing world. So it helps the health professionals to identify prolonged labor and to take appropriate actions [9].

Despite the documented benefits and recommendations, utilization of the partograph is either poor, inconsistent or used incorrectly [11, 12]. The most important barriers to use of the partograph are low-resource settings, shortage of human resources, low competence, lack of on-going facilitative supervision, acceptability of the tool and lack of functioning referral mechanisms present major challenge to effective use of the partograph [13]. A study done in Ethiopia revealed that majority (99%) of the participants knew about the partograph. However, only 21.8% of them indicated utilization of the partograph can reduce maternal and newborn mortality [14].

Previous studies [12, 14, 15] have reported levels of knowledge and utilization of the partograph in other part of Ethiopia. However, most of these studies were restricted to urban health facilities and limited geographical regions which might obscure to understand better estimates. There was a paucity of information about level of health professionals’ knowledge and use of the partograph in public health institutions in eastern Ethiopia. Therefore, we aimed to assess health professionals’ knowledge and use of the partograph at public health institutions in Eastern Ethiopia.

## Methods

### Study setting and period

The study was conducted in public health institutions from Dire Dawa city administration, Harari regional state and East Hararghe zone of Oromia regional state in Eastern Ethiopia from March 1st to April 30th, 2015. According to the Central Statistical Agency (2007), Harari regional state had 122,000 total population (60,000 were male and 62,000 were females). Harari regional state had two public hospitals and 8 health centers. During the study period Dire Dawa had a population of 341,834, of whom 171,461 were men and 170,461 women; 233,224(68.23%) of the population were urban inhabitants [16]. Dire Dawa city administration has two public hospital and 16 health centers. East Hararghe is one of the Zones of the Oromia region which has four public hospitals and 83 health centers.

### Study design and population

A cross-sectional study design with quantitative method was used to examine knowledge and utilization of the partograph. Health professionals who were working at least for six months in maternity unit at public health institutions in Dire Dawa city administration, Harari regional state and East Hararghe zone were randomly selected. The sample size was determined using a single population proportion formula [n = (Zα/_2_)^2^ P(1-P)/ d^2^; whereas, Z: significance level, P: proportion (prevalence), and d: marginal error]. We considered 95% significance level, 5% of the marginal error and proportion of partograph utilization (53.3%) obtained from study done in the Addis Ababa [15]. The minimum sample size required was estimated to be 441 using the above formula, and including 15% for non-response rate.

**Fig. 1:**
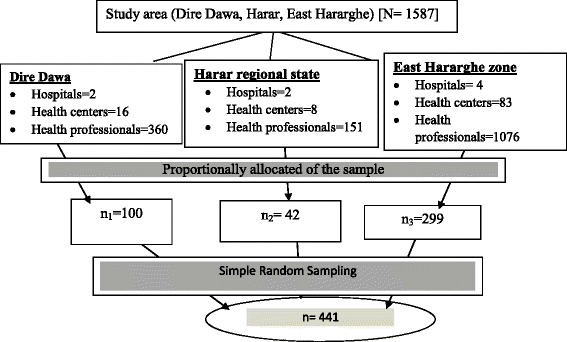
Schematic presentation of sampling procedure

The sample was proportionally allocated to the institutions to the number of health professionals working in the maternity units of the respective health institutions. Therefore, 441 health professionals were involved on this study who have been working in a sample of 56 health centers out of 107 total health centers and eight hospitals in Dire Dawa, Harar and East Hararghe zone. Eventually study participants were selected randomly through lottery method in each health institution (Fig. 1).

### Data collection and quality assurance

Data were collected using structured self-administered questionnaire about health professionals’ knowledge and utilization of the partograph. The structured questionnaire was adapted from literature [15, 17]. The questionnaire was contextualized to the local situation and research objectives. It consisted of the socio-demographic characteristics of the study participants, other variables related to routine obstetric care and the content of the partograph to be filled during labor follow up. Eight midwives who were able to speak both Amharic and Affan Oromo were recruited and trained as data collector. Pre-test was done prior to the actual data collection nearby health institutions (Gende Gerada health center in Dire Dawa and Sofi health center in Harari region) to ensure clarity, wordings, logical sequence and skipping patterns of the questions. The content of the WHO modified partograph was used directly for knowledge and utilization assessment. We assessed health professionals’ knowledge using scores for correct answers given to knowledge items (Table 1).

**Table 1:** Criteria for the partograph knowledge score

Parameters	No	Yes
Foetal heart rate	0	3
Colour of liquor	0	4
Cervical dilatation	0	3
Descent of the presenting part	0	5
Uterine contraction	0	5
Maternal blood pressure	0	2
Maternal pulse	0	1
Maternal temperature	0	1
Intravenous fluids & drugs	0	1
Moulding	0	5

The overall knowledge score ranges between 0 and 30, then respondents were classified as poor level of knowledge (0-10), fair level of knowledge (11-20) and good level of knowledge (21-30). The final scores were computed to give a composite scale with category (mean score or more = good or otherwise = poor) based on the cut-off point.

### Data processing and statistical analysis

Collected data were checked for completeness and consistency, coded and entered into Epi Info Version 3.5.1 software. Data from Epi Info Version 3.5.1 software were exported into SPSS version 22.0 statistical software for analysis. Data were explored and cleaned prior to analysis using SPSS version 22.0. Descriptive statistic was done to compute frequencies, percentages, mean, standard deviation and median of independent and dependent variables accordingly. Bivariate and multivariate logistic regression analysis were carried out to examine the relationship between independent variables and the health professionals’ knowledge and use of the partograph. Statistically significant association was declared considering adjusted odds ratio at 95% confidence interval and *p* value less than 0.05.

## Results

Out of the total 441 health professionals included to this study, 432(98%) participants gave a complete response providing a non-response rate of 2%.

### Socio demographic characteristics of the health professionals

The mean age of the respondents was 26.8(±4.1) years. More than half 228(52.8%) of the health professionals were female. The majority of health professionals 229(53.0%) were Midwives followed by Nurses 167(38.7%). Two hundred fifty nine (60%) of the health professionals were from health centers. The year of service in health care delivery was found to be ranging between less than one to 25 years with the mean duration of 4.28(±3.68) years. More than half, 222(51.4%) of them received in-service training about the partograph. Similarly, more than half 232(53.7%) of them were from urban health institutions (Table 2).

**Table 2:** Socio-demographic characteristics of the health professionals in public health institutions in Eastern Ethiopiafrom March 1st to April 30th, 2015 (*n* = 432)

Variables	Categories	Frequency	Percent
Age (in years)	20-25	178	41.2
	26-30	205	47.5
	31-35	28	6.5
	>35	21	4.9
Marital status	Single	195	45.1
	Married	214	49.5
	Divorced/widowed	23	5.4
Sex	Male	204	47.2
	Female	228	52.8
Professional qualification	Diploma Nurse	113	26.2
	Diploma Midwife	165	38.2
	B.Sc. Nurse	54	12.5
	B.Sc. Midwife	64	14.8
	Health Officer	20	4.6
	Medical Doctor (GP)	16	3.7
Type of health facility	Hospital	176	40.0
	Health center	259	60.0
Location of health facility	Urban	232	53.7
	Rural	200	46.3
Year of service	Five years and less	329	76.2
	Over five years	103	23.8
Received in-service training on partograph	Yes	222	51.4
	No	210	46.6

### Health professional’s knowledge about the partograph

The knowledge of health professionals about assessment of labor using the partograph was also investigated. Only 231(53.5%) of the health professionals indicated diagnosing foetal distress using the partograph. The majority 384(80.5%) of them knew the pattern of abnormal foetal heart rate using the partograph. Similarly, 340(78.7%) of them knew about the assessment of satisfactory progress of labor using the partograph (Table 3).

**Table 3:** Health professionals knowledge on assessment of labor using the partograph in public health institutions in Eastern Ethiopia from March 1st to April 30th, 2015 (*n* = 432)

Knowledge of assessment of labor with partograph	Frequency	Percent
Prolonged labor	257	59.5
Obstructed labor	316	73.1
Poor progress of labor	259	60.0
Inefficient uterine contraction	305	70.6
Suspected fetal distress	231	53.5
Abnormal fetal heart rate	384	80.4
Satisfactory progress of labor	340	78.7
Need of augmentation	257	63.7

From the total of 432 health professionals, only 196(45.4%) of them had fair knowledge of the partograph while 187(43.2%) and 49(11.3%) of them had good and poor knowledge respectively. One third of midwives (B.Sc.) had good level of knowledge while only one out of ten diploma nurses had good level of knowledge. Approximately three fourth 299(71%) of them reported that pre-service training was primary source of knowledge.

### Factors associated with knowledge of health professionals about the partograph

On bivariate analysis type of health institution, in-service training and professional level were the variables found to be significantly associated with knowledge about the partograph. Health professionals working in health centers were 69% more likely to have poor level of knowledge about the partograph compared to those working in hospitals [AOR = 0.31, 95% CI: 0.20, 0.47]. Health professionals who had in-service training on the partograph were two times more likely to have good level of knowledge compared to those who never had formal training [AOR = 2.0, 95% CI:1.22, 3.42]. Midwives (B.Sc.) were 2.8 times more likely to have good level of knowledge about the partograph than medical doctors. Other variables such as sex and professional tenure and attitude towards the partograph did not show any statistically significant association with the level of knowledge of the health professionals about the partograph (Table 4).

**Table 4:** The relationship between independent variables and health professionals level of knowledge about the partograph in public health institutions in Eastern Ethiopiafrom March 1st to April 30th, 2015 (*n* = 432)

Variables	Categories	Overall knowledge		COR(95% CI)	AOR(95% CI)
		Poor (%)	Good (%)		
Sex	Male	102(50)	102(50)	0.75(0.52, 1.10)	
	Female	98(43)	130(57)	1.00	
Type of institution	Health center	151(58.3)	108(41.7)	0.28(0.18,0.43)	0.31(0.20, 0.48)^*^
	Hospital	49(28.3)	124(71.4)	1.00	1.00
Location of health facility	Urban	90(38.8)	142(61.2)	1.93(1.3,2.83)	1.40(0.94, 2.13)
	Rural	110(55%)	90(45%)	1.00	1.00
Year of service	5 year and less	158(48%)	171(52)	0.75(0.48, 1.17)	
	Over 5 year	42(40.8)	61(59.2)	1.00	
In-service training	Yes	78(35.1)	144(64.9)	2.56(1.72,3.78)	2 (1.22, 3.42)^*^
	No	122(58.1)	88(41.9)	1.00	1.00
Professional qualification	Diploma Nurse	96(85)	17(15)	0.06(0.02, 0.21)	0.06(0.02,2.36)
	Diploma Midwife	66(40)	99(60)	0.50(0.16, 1.62)	1.30(0.39, 4.50)
	B.Sc. Nurse	30(55.6)	24(44.4)	0.40(0.14, 1.70)	1.54(0.43, 5.60)
	B.Sc. Midwives	2(9.4)	62(90.6)	3.20(4.23, 6.98)	2.80(1.60, 5.60)^*^
	Health officer	6(30)	14(70)	0.78(0.18, 3.42)	0.18(0.18, 3.42)
	Medical Doctor	9(56.3)	7(43.7)	1.00	1.00
Do you like partograph	Yes	78(33.8%)	153(66.2%)	3.02(2.05, 4.49)	0.47(0.30, 1.76)
	No	122(60.7%)	79(39.3%)	1.00	1.00

*P < 0.05

### Partograph utilization and associated factors

Four hundred (92.6%) of the health professionals reported that they were using the partograph to follow labor progress. Of these, 97.1% of them who were working at hospital, and 96.8% of health professionals had in-service training on how to use the partograph. Type of health institutions did not have any statistical difference in terms of health professionals’ use of the partograph [AOR = 1.50, 95% CI: 0.53, 4.38]. On the other hand, health professionals who had in-service training about the partograph were three times more likely use of the partograph than those who had not have in-service training [AOR = 3.10,95% CI:1.35, 4.98]. In addition, health professionals who had positive attitude about the partograph had higher odds of utilizing the partograph in monitoring of laboring women compared to those who lacked positive attitude towards the partograph [AOR = 2.90,95% CI: 1.30, 6.30] (Table 5).

**Table 5:** The relationship between independent variables and the partograph utilization in public health institutions in Eastern Ethiopiafrom March 1st to April 30th, 2015 (*n* = 432)

Variables	Categories	Partograph utilization		COR (95% CI	AOR (95% CI)
		Yes (%)	No (%)		
Sex	Male	187(91.7)	17(8.3)	1.29(0.63, 2.66)	
	Female	213(93.4)	15(6.6)	1.00	
Type of institution	Health center	232(89.6)	27(10.4	0.26(0.10, 0. 68)	1.50(0.53, 4.38)
	Hospital	168(97.1)	5(2.4)	1.00	1.00
In-service training	Yes	215(96.8)	7(3.2)	4.10(1.75, 9.82)	3.1(1.35, 4.98)*
	No	185(88.1)	25(11.9)	1.00	1.00
Year of service	Five years and less	303(92.1)	26(7.6)	0.72(0.29, 1.8)	
	Over five years	97(94.2)	6(5.8)	1.00	
Do you like partograph	Yes	229(99.1)	2 (0.9)	20.0(4.74, 85.20)	2.90 (1.30, 6.30)*
	No	171(85.1)	30(14.9)	1.00	1.00

*P < 0.05

## Discussion

This study tried to find out the level of the health professionals knowledge and use of the partograph in public health institutions in eastern Ethiopia. Almost all of respondents knew what a partograph was even though only less than half of subjects had good level of knowledge about the partograph. This study finding implies that health professional’s knowledge about the partograph might be inadequate for better utilization of the partograph in public health institutions. This study finding is consistent with previous studies done in Ethiopia and in Nigeria [11, 15].

The result revealed existence of significant association between type of health institutions and level of health professionals’ knowledge about the components of the partograph. The odds of having a good knowledge about components of the partograph was higher in obstetric care givers working in health centers than hospitals. However, the result was not consistent with other studies done in Addis Ababa (Ethiopia) [15]. This might be because of health professionals who were working in hospitals had extra burden and being busy with client flow.

Presence of in-service training on obstetric care was one of the associated factors with the health professional’s knowledge about the partograph. Health professionals who received training were better be able to explain components of the partograph. This finding may support the idea that training improves the status of existing health professionals’ knowledge about the partograph. Still this finding is consistent with previous studies done in, Nigeria and Ethiopia [11, 14, 15]. This finding points to the need that health professionals should get regular in-service refresher trainings on the obstetric care.

In this study, finding on the health professionals’ overall knowledge of obstetric care was found to be good, and about 53.7% of them had a mean score level of knowledge of the partograph. Midwives (B.Sc.) were more likely to have good knowledge than general medical practitioners. This might be explained by the fact that they might have a better chance of exposure to obstetric training and practice than general medical practitioners. However, this finding is inconsistent with the study done in Ethiopia which showed general medical practitioners had a better knowledge about the partograph than midwives [14]. This finding emphasizes the need for general medical practitioners to be considered equally to receive in-service obstetric care training.

In this study, the utilization of the partograph was significantly higher among health professionals. This is consistent with the study done in Ethiopia [15]. The result of this study also revealed existence of significant association between participants’ attitude towards the partograph and utilization of it. This is in agreement with the studies done in Nigeria and Ethiopia [14, 15, 18], that support the idea that the partograph is an efficacious tool for monitoring labor and identify women requiring further interventions.

Additionally 59.5% of the respondents in our study knew how to diagnosis the presence of prolonged labor using the partograph and 78.7% of them knew about the assessment of satisfactory progress of labor. This is in-line with the finding that showed the partograph has been indicated as the need to address prolonged and poor progress of labor [8]. This study has its own strength and limitations. We tried to include health professionals from health centers and hospitals and inclusion of private health professionals would have given comprehensive picture and make generalization possible. However, findings from this study can be regarded as a snapshot of current knowledge and practice of the partograph utilization within the study area. In addition, assessment of utilization was solely based on self-report of health professionals which might overestimate the finding.

## Conclusions

This study revealed that only less than half of health professionals (obstetric care givers) had fair knowledge about the partograph in public health institutions in eastern Ethiopia. Presence of in-service obstetric care training, type of health institution and profession were the variables found to be significantly associated with knowledge of the partograph. Lack of training for health professionals and lack of positive attitude towards the use of the partograph were significantly related to the utilization of the partograph. Periodic on-job (in-service) training of health professionals on the use of the partograph, regular supportive supervision, motivating staffs to utilize the partograph and record their findings should be given emphasis.

## Abbreviations

AOR: Adjusted odds ratio
CI: Confidence interval
COR: Crude odds ratio
WHO: World Health Organization
